# Macrophages Derived From Human Induced Pluripotent Stem Cells Are Low-Activated “Naïve-Like” Cells Capable of Restricting Mycobacteria Growth

**DOI:** 10.3389/fimmu.2020.01016

**Published:** 2020-06-04

**Authors:** Tatiana Nenasheva, Tatiana Gerasimova, Yana Serdyuk, Elena Grigor'eva, George Kosmiadi, Alexander Nikolaev, Erdem Dashinimaev, Irina Lyadova

**Affiliations:** ^1^Laboratory of Cellular and Molecular Basis of Histogenesis, Koltzov Institute of Developmental Biology of the Russian Academy of Sciences, Moscow, Russia; ^2^Laboratory of Biotechnology, Department of Immunology, Central Tuberculosis Research Institute, Moscow, Russia; ^3^Laboratory of Developmental Epigenetics, Federal Research Center Institute of Cytology and Genetics, The Siberian Branch of the Russian Academy of Sciences, Novosibirsk, Russia; ^4^Center for Genome Technologies, Pirogov Russian National Research Medical University, Moscow, Russia; ^5^Laboratory of Cell Biology, Koltzov Institute of Developmental Biology of the Russian Academy of Sciences, Moscow, Russia

**Keywords:** induced pluripotent stem cell derived macrophages, M1 and M2 macrophages, CD16, HLA-DR, *Mycobacterium tuberculosis*, cytokines, chemokines

## Abstract

In peripheral tissues, immune protection critically depends on the activity of tissue resident macrophages, which makes our understanding of the biology of these cells of great significance. Until recently, human macrophage studies were largely based on the analysis of monocyte-derived macrophages that differ from tissue resident macrophages by many characteristics. To model tissue resident macrophages, methods of generating macrophages from pluripotent stem cells have been developed. However, the immunological properties of macrophages derived from pluripotent stem cells remain under-investigated. In this study, we aimed to perform the multifarious immunological characteristics of macrophages generated from human induced pluripotent stem cells (iMϕs), including an analysis of their phenotype, secretory and antibacterial activities, as well as their comparison with macrophages derived from blood monocytes and infected lung tissue. We report that iMϕs displayed the morphology and the CD11b^+^CD45^+^CD14^+^ phenotype typical for mononuclear phagocytes. The cells co-expressed markers known to be associated with classically (CD80, CD86, CCR5) and alternatively (CD163 and CD206) activated macrophages, with a bias toward a higher expression of the latter. iMϕs secreted pro-inflammatory (IL-6, CXCL8, CCL2, CCL4, CXCL1, CXCL10) and anti-inflammatory (IL-10, IL-1RA, CCL22) cytokines with a high IL-10/IL-12p70 index (>20). iMϕs were phagocytic and restricted *Mycobacterium tuberculosis* growth *in vitro* by >75%. iMϕs differed from blood monocytes/macrophages by a lower expression level of HLA-DR and the CD14^+^CD16^int^ phenotype and shared several phenotypic characteristics with lung macrophages. In response to LPS, iMϕs up-regulated HLA-DR and produced TNF-α. IFN-γ increased iMϕ reactivity to LPS, but did not increase iMϕ mycobactericidal capacity. The results characterize iMϕs as differentiated but low-activated/low-polarized “naïve-like” macrophages that are capable of mounting inflammatory and antibacterial responses when exposed to inflammatory stimuli or pathogens. iMϕs represent a valuable model for studying antibacterial responses of tissue resident macrophages and for developing approaches to modulating macrophage activity.

## Introduction

Macrophages play a key role in host homeostasis by regulating the immune response, eliminating pathogens and damaged self-cells, as well as repairing tissues after injury and infection. A dysregulation of macrophage function can cause infections, chronic inflammation and violation of tissue structure, and it is involved in the pathophysiology of many disorders, such as cancer, neurodegenerative and cardiovascular diseases (1–4). It is accepted that macrophages represent an attractive intervention target for the treatment of various pathologies (1, 5, 6). Yet, to develop a macrophage-oriented cell therapy, an in-depth understanding of macrophage biology is needed.

Macrophages execute their functions locally, in the tissues where they reside. It has long been accepted that tissue macrophages originate from circulating adult blood monocytes that mature into macrophages once they have migrated to the tissues (7–9). A series of recent experimental mouse studies have challenged this concept by demonstrating that tissue resident macrophages (TRMs) develop from embryonic progenitors that seed the tissues during the embryonic and early postembryonic periods (10–14). New concepts have arisen that regard TRMs as differentiated long-lived cells capable of proliferating and self-renewing independently of bone marrow/blood-derived monocytes. These concepts do not assign a significant role to blood-derived monocytes/macrophages in the maintenance of tissue macrophage pool (14–17). Nevertheless, the input of monocytes into the tissue macrophage pool is different in different tissues and conditions. Particularly, in the gastrointestinal tract, skin and heart, embryonic macrophages are replaced with age by cells deriving from circulating monocytes; in other tissues, the migration of blood monocytes is promoted by inflammation (17–21). Thus, another view is that blood monocytes can permanently replace embryonic TRMs in the tissues (22, 23).

Notably, most of our knowledge of macrophage ontogeny comes from studies of inbred mice that allow performing fine-tuned experiments, including cell transfer, parabiosis, the tracing of gene-manipulated cells or depletion of specific cell populations. These approaches, however, may not properly reflect the processes that go on in humans: cell and gene manipulations disturb macrophage homeostasis and may change their migratory and self-renewal properties; specific-pathogen-free conditions, a constant diet and other mouse housing factors affect macrophage turnover (e.g., by dampening the baseline level of inflammation or by producing stress (22, 24); macrophages derived from different species and even from different mouse strains display significant differences in their gene expression profiles and functionality (22, 25–27). Thus, the extent to which the data obtained in manipulated inbred mice model the processes going on in humans remains unclear; there is no consensus view on the origin and fine characteristics of human TRMs, and there is a need for new models to address these questions.

Independently of their origin, in the tissues, macrophages are permanently exposed to tissue-specific, microenvironmental and inflammatory stimuli that shape macrophage morphology, gene expression and function and create a high degree of their heterogeneity (20, 28–31). In spite of this heterogeneity, until recently, only a limited number of approaches were available to study macrophage biology. These included human and mouse cell lines, macrophages derived from the bone-marrow or different tissues of experimental animals and human blood monocyte-derived macrophages (MDMs), which only partly can model human TRMs (32, 33).

In response to a need for a better *in vitro* model of human macrophages, methods of their generation from pluripotent stem cells, either embryonic or induced (iPSCs), have recently been elaborated [reviewed in (34)]. The methods are based on a stepwise differentiation of pluripotent stem cells into hemogenic cells, monocyte-like cells (iMCs) and macrophages (iMϕs). In most protocols, the differentiation is driven by growth factors and cytokines that are sequentially added to cell cultures, such as bFGF, BMP4, activin A, VEGF (all induce hemogenic endothelial specification and endothelial-to-hematopoietic transition); IL-6, SCF, IL-3 (these promote the expansion of hematopoietic progenitors); CSF1 (also called M-CSF, induces monocytic differentiation) (35–38). Recently, simplified methods for iMϕ generation have been suggested. The methods are based on the spontaneous formation of embryoid bodies (EBs, i.e., three-dimensional aggregates of iPSCs able to differentiate in different directions) and their monocytic differentiation driven by only two factors, IL-3 and CSF1, which makes the methods less time- and resource-consuming (39, 40).

The use of either of the protocols of iMϕ generation results in the formation of cells that display macrophage-like morphology, express pan-macrophage markers (i.e., CD45, CD11b, CD14 in humans and CD11b and F4-80 in mice) and are phagocytic, the triad of traits that in all iMϕ studies is used to confirm cell macrophage nature (37, 39–43). More in-depth characteristics of iMϕs were performed by several groups. Phenotypic analyses demonstrated the expression of CD163, CD206, MHC class II, CD40 and several other markers by iMϕs (40, 44–46). However, different authors used different sets of markers, and the levels of marker expression differed between the studies, leaving the iMϕ phenotype not fully characterized. Transcriptomic analyses compared gene expression profiles of iMϕs and MDMs, demonstrated their global similarities, but also revealed significant differences, particularly, in the expression of genes associated with antigen presentation (lower in iMϕs) and tissue remodeling [higher in iMϕs (36, 42, 47)]. Takata and co-authors showed transcriptomic similarity of mouse iMϕs and yolk sac macrophages and different transcriptomic features of bone-marrow derived macrophages (46). Buchrieser and co-authors demonstrated that human iMϕs share ontogeny with *Myb*-independent TRMs (43). It was suggested that compared to MDMs, iMϕs are biased toward more primitive cells, that the process of iMϕ differentiation recapitulates embryonic hematopoiesis and that iMϕs model TRMs (34, 43, 46). Yet, the type and tissue identity of macrophages generated from pluripotent stem cells are not fully understood (34).

Functional analyses of iMϕs largely focused on the examination of their responsiveness to inflammatory stimuli, particularly, to IFN-γ/LPS and IL-4. The stimuli are known to induce the “polarized” activation of macrophages resulting in the formation of cells with pro- (IFN-γ/LPS-stimulated) and anti- (IL-4-stimulated) inflammatory activities. The cells were classified as “classically” and “alternatively” activated, respectively, or “M1” and “M2” [(48–54) and see (55) for the critical discussion of macrophage nomenclature]. Later studies revealed time-, dose-, and stimulus-dependent transcriptomic differences within the “M1” population (56–59) and identified several “subpopulations” of M2 macrophages (52, 53, 60). Macrophages exposed to various other stimuli were analyzed and shown to express a continuum of activation states that did not fall into the “M1/M2” paradigm (26, 55, 58, 61–63). Thus, the paradigm has been revised and replaced by a new “spectrum” or “multidimensional” model of macrophage activation, which takes into account macrophage capacity to respond to their local microenvironment by developing a wide range of various transcriptional programs (55, 58, 64) and acquiring an “endless set of phenotypes” (65). To describe macrophage activation in a unified manner, a new macrophage activation nomenclature based on the indication of the way used for macrophage activation was proposed [e.g., M(Ig), M(LPS), M(IL-4); (55)]. Yet, until now, many macrophage studies adhere to the M1/M2 paradigm and terminology, which is particularly true for studies analyzing iMϕs (e.g., 36, 37, 40). Thus, for consistency purposes, in this paper, the M1/M2 terminology is kept when discussing these studies. iMϕs were shown to respond to both M1 (IFN-γ/LPS) and M2 (IL-4) polarizing stimuli and to be more reactive to the former, which can result from an initial bias of iMϕs toward an M2 type (36, 37, 39, 40). Nevertheless, the baseline pattern of cytokine production by iMϕs needs further investigation.

The other key functional property of macrophages is their antibacterial activity. Although inflammatory and antibacterial responses are interrelated, they are controlled by different classes of genes (66), indicating that the antibacterial potential of iMϕs requires a separate investigation. iMϕs can be infected with intracellular pathogens, including *Chlamydia trachomatis* (67), HIV (39), *Salmonella* (41), and *Mycobacterium tuberculosis* (68). However, the extent to which iMϕs are able to control bacterial growth remains unclear. In the study by Yeung et al. (67), iMϕs supported the entire life cycle of *C. trachomatis*. Han and co-authors reported that iMϕs are permissive for *M. tuberculosis* (68). On the other hand, Hale and coauthors showed that iMϕs were able to kill *Salmonella Typhi* and *S. Typhimurium* (41). In the study by Ackermann et al., iMϕs restricted *Pseudomonas aeruginosa* growth *in vitro* and even rescued mice from acute infection mediated by *P. aeruginosa* at the lower respiratory tract suggesting iMϕs as a promising approach for the immunotherapy of infectious diseases (69). Thus, more investigations are needed to unravel iMϕ activity toward various pathogens.

In this study, we aimed to perform a multifarious analysis of iMϕ phenotype, secretory and antimycobacterial properties, as well as to compare their characteristics with those of monocyte-derived and lung tissue residing macrophages. We report that iMϕs are low-activated functionally unbiased cells that: (i) co-express markers associated with M1 [i.e., M(IFN-γ) and M(LPS)] and M2 [i.e., M(IL-4)] activation; (ii) co-produce pro- and anti-inflammatory factors; (iii) are reactive to inflammatory stimuli; (iv) are able to restrict mycobacterial growth; (v) are phenotypically similar (although not identical) to MDMs and share several phenotypic characteristics with macrophages isolated from the infected human lung. The results provide detailed phenotypic and functional characterization of iMϕs and suggest that iMϕs represent a useful model to study human macrophage immunity and cell-pathogen interactions.

## Materials and Methods

### Human iPSC Culture and EB Formation

If not indicated otherwise, iMϕs were differentiated from the iPSC line called iMA generated from human embryonic dermal fibroblasts (70). The main results were verified using iMϕs differentiated from the human iPSC line called KYOU-DXR0109B [201B7, ATCC® ACS-1023™] (KYOU). iPSCs were expanded by culturing them onto mouse feeder cells in a 35 mm diameter Petri dish (~5 × 10^5^ cells/dish) in Knockout DMEM (cat #10829018, Gibco, Invitrogen, Carlsbad, CA, USA) supplemented with 15% Knockout serum replacement (cat #10828028, Gibco), 0.055 mM β-mercaptoethanol (cat #M3148, Sigma-Aldrich, St. Louis, USA), 2 mM L-Glutamine (cat #A2916801, Gibco), 1% non-essential amino acids (NEAA, cat #11140050, Gibco), 1% pyruvate Na (cat #11360070, Gibco), 1% penicillin/streptomycin (cat #15140122, Gibco) (iPSC/EB culture medium) and 10 ng/ml basic fibroblast growth factor (bFGF, cat #710304, Biolegend, San Diego, CA, USA). Feeder cells were obtained from 12-days mouse embryos, treated with mitomycin C (10 mg/ml, cat #M4287 Sigma Aldrich) and stored at −80°C until the use. The medium in iPSC cultures was replaced daily. When the cells reached 70–80% confluence, they were used to generate EBs. For that, iPSC colonies were treated with Collagenase IV (1 mg/ml, 5–20 min; cat #17104019, Gibco), washed and cultured in ultra-low adhesive 6-well plates (cat # 3471, Corning Inc., Corning, NY, USA) in iPSC/EB culture medium until large (200 μm or more in diameter) EBs formed (4–6 days). The medium was replaced daily; for the first 24 h of culture, Rock Inhibitor (Y-27632, cat #SCM075, Sigma-Aldrich) was added.

### iMC Differentiation and Maintenance

iMCs were generated using the protocol described by van Wilgenburg and co-authors with some modifications (39). Briefly, EBs were selected, manually transferred onto 6-well tissue culture plates (20–30 EBs/well, cat #3516, Corning Inc.) and cultivated in X-VIVO 15 medium (cat #BE04-744Q, Lonza, Bazel, Swiss) supplemented with 2 mM Glutamax (cat #35050061, Gibco), 1% penicillin/streptomycin, 0.055 mM β-mercaptoethanol (iMC/iMϕ differentiation medium) in the presence of 25 ng/ml IL-3 and 100 ng/ml CSF1 (cat #578006 and #574804, both from Biolegend). Full medium change was performed every 7 days.

**Figure 1:**
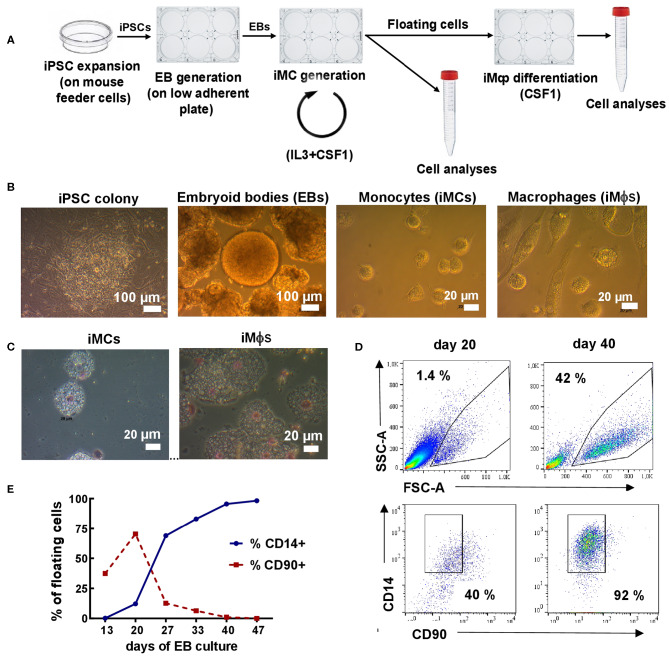
Differentiation of iMϕs from iPSCs. **(A)** Workflow of iMϕ generation. iPSCs were expanded on mouse feeder cells and transferred into low-adherent plates to generate EBs. EBs were cultured in the presence of IL-3 and CSF1 to allow the generation of iMCs. Starting day 15–20, floating cells appeared in the cultures. The cells were collected and used for: (i) terminal differentiation into iMϕs; (ii) flow cytometry, morphological and functional analyses. The remaining adherent cells were cultured in IL-3/CSF1 containing medium to continue iMC generation. The procedures were repeated every 7 days. **(B)** Representative light microscopy of differentiating cells. **(C)** Cytospins of iMCs and iMϕs. **(D)** Representative flow cytometry analysis of floating cells (days 20 and 40). **(E)** The accumulation of CD14^+^ and the disappearance of CD90^+^ cells in the cultures. Representative results of one out of seven independent experiments.

When floating monocyte-like cells appeared in the cultures, they were collected, counted and used for: (i) phenotypic and other types of analyses; (ii) differentiation into macrophages. The remaining adherent cells were fed with a new iMC/iMϕ differentiation medium to maintain iMC differentiation (Figure 1A). The procedures were repeated every 7 days.

### iMC Differentiation Into iMϕs

iMCs generated in EB cultures were collected and cultured in iMC/iMϕ differentiation medium supplemented with CSF1 (100 ng/ml) for 7 days. Depending on the aims of the experiment, the cells were cultured in 6-, 24- or 96-well plates (5–8 × 10^5^, 2–2.5 × 10^5^, and 3–4 × 10^4^ cells/well, respectively). In some experiments, iMCs were cleansed of antibiotics prior to their differentiation into iMϕs, by double washing in an antibiotic-free medium. The cells were then cultured in iMC/iMϕ differentiation medium deprived of antibiotics.

### Blood Monocyte Isolation and Differentiation Into MDMs

Venous blood samples were obtained from healthy donors who gave written informed consent under the protocol approved by the IRB#1 of CTRI. The samples were collected in heparin tubes; peripheral blood mononuclear cells (PBMCs) were isolated within 1 h of sample collection by Ficoll density gradient centrifugation (1.077, cat #1714400, GE Healthcare, NY, USA). To generate MDMs, monocytes (CD14^+^ cells) were isolated from PBMCs using human CD14 MicroBeads kit (cat #130-050-201, Milteny Bergisch Gladbach, Germany) and cultured in the presence of CSF1 (100 ng/ml) for 7 days.

### Lung Tissue Cells

Lung cells were obtained from lung tissue surgically resected from patients with diagnosed pulmonary tuberculosis (TB). Three patients were enrolled in the study (2 men, one woman; 27, 28 and 30 years old). Written informed consent was obtained from all enrolled patients, and the study was approved by the IRB#1 of CTRI. The samples of lung tissue were obtained within 30 min of tissue resection so as to avoid the inclusion of the grossly fibrotic wall of the cavity. Tissue samples were immersed in ice-cold RPMI 1640 medium (cat #11875-093) supplemented with 5% FCS (cat #A31605), 20 U/ml heparin, and 50 mg/ml gentamicin (cat #15750060, #H3149 all from Thermo Fisher Scientific, Waltham, MA, USA), rinsed to remove blood clots, carefully minced with a sterile surgical blade, chopped with scissors and pipetted. The resulting suspensions were filtered through a 100-mm-mesh stainless steel sieve, double-washed and enriched for mononuclear cells using Ficoll density gradient centrifugation. From the same patients, blood samples were obtained on the same day and PBMCs were isolated. Lung tissue-derived cells and PBMCs were simultaneously treated with PE-anti-CD206 (clone 3.29B1.10, Beckman Coulter, USA), PerCP-Cy5.5-anti-CD14 (clone MΨP-9), APC-anti-HLA-DR (clone L243), BV421-anti-CD11b (clone ICRF44) and BV510-anti-CD16 (clone 3G8) antibodies (all from BD Bioscience, San Jose, CA, USA) and analyzed by flow cytometry using the same instrument settings.

### Flow Cytometry

Cells (3 × 10^5^/sample) were stained with PerCP-Cy5.5-anti-CD14 (clone MϕP9) along with FITC-anti-CD45 (clone hi30), PE-anti-CD90 (clone 5E10, eBioscience, an Affymetrix company, Vienna, Austria), APC-anti-CD34 (clone 8G12), BV421-anti-CD11b (clone ICRF44) and BV510-anti-CD16 (clone 3G8) or FITC-anti-CD64 (clone 10.1), PE-anti-CD206 (clone 3.29B1.10), APC-anti-HLA-DR (clone L243), BV421-anti-CD163 (clone ghi/61) and BV510-anti-CD86 (clone 2331 (FUN-1) or FITC-anti-CD80 (clone L307.4), PE-anti-CD195 (clone 2D7/CCR5) and BV510-anti-CD86 (all from BD Bioscience, San Jose, CA, USA). The cells were washed, fixed and analyzed on BD FACS Canto II (BD Biosciences) using FACSDiva™ (BD Biosciences) and FlowJo (TreeStar) software. Unstained, isotype, single-stained and fluorescence-minus-one (FMO) controls were included. For each marker, isotype controls were used in at least one experiment and were shown not to differ significantly from negative controls. In contrast, single-staining with anti-CD14 antibodies affected the level of the “expression” of several markers. Therefore, for all markers, negative populations were determined using CD14-single-stained or FMO control. Single-stained controls were also used to verify the adequacy of the automatic compensations. Optimal instrument settings for macrophage analysis were determined in preliminary experiments; the same settings were then used throughout all the experiments.

### Microscopy and Cytospins

Light images were obtained using a Zeiss Axioskop 40 microscope equipped with an AxioCamMRc5 camera (Carl Zeiss, Germany). Cytospins were prepared using a Shandon cytocentrifuge (ThermoScientific, Langenselbold, Germany). Briefly, 3–4 × 10^3^ cells were spun for 3 min at 1,000 rpm. Slides were dried, treated with methanol for 5–10 min, stained with Romanovsky-Giemsa stain (Merck, Germany) for 15–30 min, dried and analyzed using bright-field microscopy.

### Phagocytic Activity

Phagocytosis was assessed using the commercial Phagotest™ kit (cat #341060, BD Bioscience) according to the manufacturer's instructions. Briefly, FITC labeled *E. coli* was added to 3 × 10^5^ tested cells resuspended in 200 μl of medium supplemented with 50% FCS. The suspensions were placed on ice for 10 min followed by the incubation at 37°C (test sample) or 0°C (negative control) for 30 min. The assay was stopped by adding 100 μl of quenching solution; the samples were washed and analyzed by flow cytometry.

### Antimycobacterial Activity

*Mycobacterium tuberculosis* (*Mtb*) strain H37Rv were prepared for macrophage infection as described previously (71). iMϕs were generated from iMCs in antibiotic-free iMC/iMϕ differentiation medium. The cells were plated in the wells of a flat-bottom 96-well plate (3–4 × 10^4^ cells/well) in RPMI-1640 medium supplemented with 2% FCS, 2 mM L-Glutamine, 1% HEPES, 1% sodium pyruvate and 0.055 mM β-mercaptoethanol, and were allowed to adhere (37^0^C, 1 h). In some experiments, CSF1 (100 ng/ml) and/or IFN-γ (100 U/ml; cat #570204 Biolegend) were added to the cell medium. *Mtb* were added to iMϕs at the multiplicity of infection of 5. The cells were incubated at 37°C, 5% CO_2_ for 3 days. For the last 18 hours, [3H]-uracil was added to the cultures, and *Mtb* growth was assessed by measuring [3H]-uracil uptake as described previously (34). Cultures containing only *Mtb* or iMϕs without *Mtb* were used as positive and negative controls, respectively. Percent inhibition of *Mtb* growth was calculated as: 100—(cpm in experimental well—cpm in negative control) × 100/cpm in positive control (where cpm is [3H]-uracil count per minute).

To determine actual numbers of *Mtb* colony forming units (CFUs), in some experiments, cells were harvested, lysed in sterile water, serially diluted and plated onto Dubos agar (cat #BD 238510, Difco™ Becton, Dickinson and Company, Franklin Lakes, NJ). *Mtb* CFUs were calculated 21 days later.

### Multiplex Analysis, ELISA and Cell Stimulation

Supernatants were obtained from iMϕ and MDM cultures, aliquoted and stored at −20°C until the day of analysis. Cytokines were determined using MILLIPLEX MAP Human Cytokine/Chemokine kit (cat #HCYTOMAG-60K, EMD Millipore Corp., MA, USA) that evaluates the following 41 factors: EGF, CCL11 (eotaxin), CSF2 (GM-CSF), CSF3 (G-CSF), IFNα2, IFNγ, IL-10, IL-12P40, IL-12P70, IL-13, IL-15, IL-17A, IL-1RA, IL-1α, IL-1β, IL-2, IL-3, IL-4, IL-5, IL-6, IL-7, CXCL8 (IL-8), CXCL10 (IP-10), CCL2 (MCP-1), CCL3 (MIP-1α), CCL4 (MIP-1β), CCL5 (RANTES), TNFα, TNFβ, VEGF, FGF-2, TGF-α, FLT-3L, CXC3CL1 (fractalkine), CXCL1 (GRO1), CCL7 (MCP-3), CCL22 (MDC), PDGF-AA, PDGF-AB/BB, sCD40L, and IL-9. All the procedures were done in accordance with the manufacturer's recommendations.

To examine iMϕ reactivity to inflammatory stimuli, cells were stimulated with *E. coli* LPS (100 ng/ml, cat #L4391, Sigma Aldrich) and/or IFN-γ (20 ng/ml) for 24 h at 37°C. Culture supernatants were analyzed using Human TNF-α ELISA assay (cat # P3H 2017/5961, Vector-Best, Novosibirsk, Russian Federation) according to the manufacturer's instructions using microplate spectrophotometer Thermo Scientific™ Multiscan™ GO and ScanIt™ Software (Thermo Fisher Scientific, Waltham, Ma, USA).

### Statistical Analysis

Data are shown as mean±SEM of at least three independent experiments. Differences between the groups were analyzed using the non-parametric Mann-Whitney test. For multiple group comparisons, Kruskal-Wallis test was used; the false discovery rate (FDR) was controlled using the Benjamini-Hochberg method with FDR set at q=0.05 (GraphPad Software Inc., San Diego, CA) (72).

## Results

### The Generation of Macrophages From iPSCs

iMϕs were generated from human iPSCs through the formation of EBs. For that, iPSCs were expanded and transferred to low-adherent plates to generate EBs; EBs were transferred to tissue-culture plates and cultured in the presence of IL-3 and CSF1 (Figure 1A). Starting days 15–20, round-shaped floating cells appeared in the cultures. The cells were large (15–20 μm in diameter), vacuolated and equipped with pseudopodia (Figures 1B,C), i.e., they resembled monocytic cells (iMCs). Once floating cells appeared in the cultures, they were harvested and used for: (i) terminal differentiation into iMϕs in CSF1 containing medium (7 days); (ii) phenotypic and other types of analyses. The remaining adherent cells were fed with a new IL-3/CSF1 containing medium to pursue iMC generation.

To characterize the floating cells, we first analyzed their expression of monocytic (CD14) and stem cell (CD90, CD34) markers. CD14^+^CD90^−^CD34^−^ cells were detectable as soon as the floating cells appeared in the cultures. The percentages of CD14^+^ cells were initially low (10-15%), but gradually increased and by day 25–40 reached 90–95% of all floating cells (Figures 1D,E). The percentages remained steadily high following further cultivation. In contrast, the numbers of floating cells increased initially, peaked between weeks 4-6, but declined afterwards. As a result, the weekly productivity of the cultures calculated as a total yield of CD14^+^ cells per well of 6-well plate per week declined after week 6 (4.0 × 10^5^± 2.6 × 10^5^cells/well at week 4, 1.9 × 10^5^±0.7 × 10^5^cells/well at week 7 and even less at later time-points).

Following terminal differentiation of iMCs into iMϕs, the cells enlarged, elongated and acquired plastic adherence (Figures 1B,C). Phenotypically, the cells retained the CD14^+^CD90^−^CD34^−^ phenotype (not shown).

### iMϕs Express the Phenotype of Differentiated but Low-Polarized Macrophages

**Figure 2:**
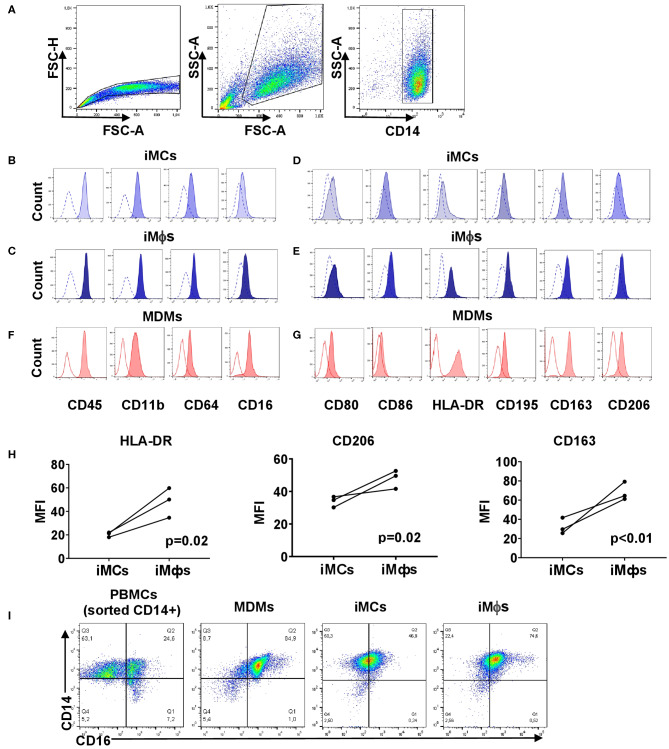
Phenotypic analysis of iMCs, iMϕs and MDMs. iMCs and iMϕs were differentiated as described in the legend to Figure 1. MDMs were obtained by isolating CD14^+^ cells from PBMCs and cultured in the presence of CSF1 for 7 days. The cells were stained with antibodies and analyzed by flow cytometry. The same instrument settings were applied for the analyses of all cells. Open histograms depict FMO control. **(A)** Gating strategy. **(B,C,F)** Surface expression of myeloid markers. **(B)** iMCs; **(C)** iMϕs; **(F)** MDMs. Representative data from one out of 5 (iMϕs) or 3 (iMCs, MDMs) independent differentiation experiments. **(D,E,G)** Surface expression of markers associated with macrophage differentiation and M1/M2 polarization. **(D)** iMCs, **(E)** iMϕs, **(G)** MDMs. Representative data from one out of at least 3 independent differentiation experiments. **(H)** Changes in the expression of HLA-DR, CD206, and CD163 following the differentiation of iMCs into iMϕs. The results of three independent differentiation experiments (mean±SEM; Mann-Whitney test). **(I)** The pattern of CD14/CD16 co-expression by PBMCs, MDMs, iMCs and iMϕs.

We then set to characterize the phenotype of iMCs and iMϕs in more details. For that, we determined the expression of receptors that characterize cell hematopoietic/myeloid nature, differentiation/maturation and polarization states. Both iMCs and iMϕs expressed CD14, CD45, CD11b and CD64 markers characteristic of mononuclear phagocytes (Figures 2A–C). iMCs expressed CD80 and CD86 (indicators of macrophage polarization toward an M1 subtype), but at low level. iMCs also expressed CD163 and CD206 (indicators of M2 macrophages), and CD195 and HLA-DR (markers of mature/activated macrophages, and also markers of M1 macrophages, Figure 2B) (73–75). Following the differentiation of iMCs into iMϕs the expression of CD163, CD206, CD195, and HLA-DR increased (*p* < 0.05), whereas the expression of CD80 and CD86 did not change (Figures 2D,E,H). The same pattern of surface marker expression was registered when iMCs and iMϕs were obtained at different points in time during the differentiation process (data not shown). Thus, the differentiation of iMCs into iMϕs was accompanied by an increase in the expression of markers that in the literature were associated with the M2 subtype (CD163, CD206) and cell maturation/M1 polarization (CD195, HLA-DR). Markers that were associated exclusively with the polarization toward M1 (i.e., CD80, CD86) were not upregulated on iMϕs. Thus, based on the expression of markers that in the M1/M2 paradigm are used to categorize macrophage subpopulations, iMϕs did not fall into either M1 or M2 subtype.

### iMϕs Differ From MDMs by a Lower Expression of HLA-DR and the Pattern of CD14/CD16 Co-expression

We then compared the phenotype of iMϕs with that of MDMs that were differentiated *in vitro* with the use of the same factor (CSF1). Both populations exhibited similar expression levels of CD45, CD80, CD163 and CD206 (Figures 2C,E–G). CD11b and CD86 were expressed at statistically higher levels on iMϕs (*p* < 0.01; Figures 2C,E–G). In contrast, the expression of HLA-DR was steadily lower on iMϕs compared to MDMs (*p* < 0.0005, Figures 2E,G), allowing considering the HLA-DR^dim^ phenotype as a characteristic feature of iMϕs.

Based on the co-expression of CD14 and CD16, blood monocytes are divided into classical CD14^+^CD16^−^, intermediate CD14^+^CD16^+^ and non-classical CD14^low^CD16^+^ subpopulations (39). We identified all three subpopulations within blood monocytes (Figure 2I). In contrast, MDMs were mostly CD14^+^CD16^+^ with a small proportion of CD14^+^CD16^−^ cells. iMCs and iMϕs appeared as one CD14^+^CD16^int^ population that differed from CD14^+^CD16^+^ MDMs by a lower level expression of CD16 (Figure 2I). The specific pattern of surface marker expression displayed by iMϕs derived from the iPSC cell line iMA was confirmed using iMϕs derived from another iPSC cell line, KYOU (data not shown).

Overall, phenotypic analysis revealed general similarities between iMϕs and MDMs, but also some differences. The latter could be due to the inter-individual variations of cells derived from different subjects or to specific features of iPSC-derived macrophages (see Discussion).

### iMϕs Share Several Phenotypic Traits With Macrophages Isolated From *Mtb*-Infected Lungs

It has been recently suggested that iPSC-derived macrophages recapitulate TRMs of embryonic origin (34). However, direct comparison of human iMϕs and TRMs is challenging due to a poor availability of the latter. This is particularly true for the lung tissue, as the main source of human lung-associated cells is bronchoalveolar lavage (BAL), and BAL macrophages do not recapitulate interstitial macrophages (76). To perform a comparative phenotypic analysis of iMϕs and lung residing macrophages, we took advantage of the availability of lung tissue surgically resected from TB patients. Lung tissue was used to prepare cell suspensions and analyze the phenotype of CD14^+^ lung cells (hereafter termed “TB-lung macrophages”). Before preparing cell suspensions, lung tissue samples were carefully rinsed to remove blood cells. However, there was a possibility that blood cells were not removed completely and that CD14^+^ “lung macrophages” contained a proportion of monocytes residing in the pulmonary blood vessels. To assess this possibility, blood samples were taken from the same patients at the day of lung surgery, and blood monocytes were phenotyped along with lung cells.

**Figure 3:**
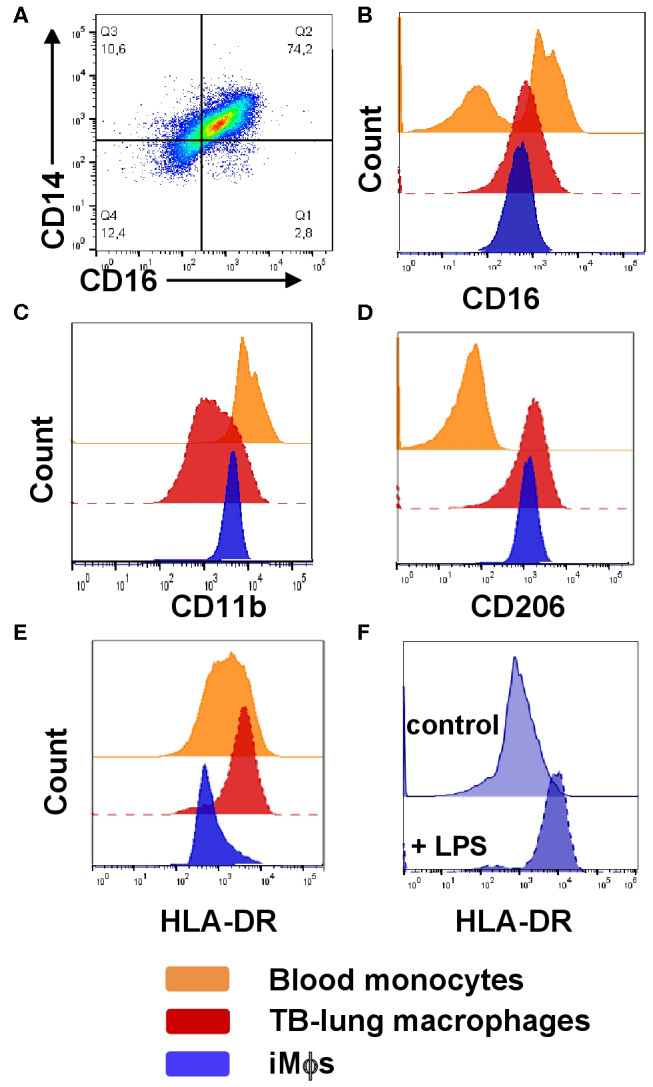
Phenotypic comparison of iMϕs with lung macrophages and blood monocytes isolated from TB patients. iMϕs were differentiated as described in the legend to Figure 1. Lung cell suspensions were prepared from the lung tissue surgically resected from TB patients as part of disease therapy. PBMCs were obtained from the same TB patients on the day of surgery. **(A)** The co-expression of CD14 and CD16 by lung tissue macrophages (gated on CD14^+^ cells). **(B–E)** The expression of CD16 **(B)**, CD11b **(C)**, CD206 **(D)**, and HLA-DR **(E)** by blood monocytes (orange), lung macrophages (red) and iMϕs (dark blue; all gated on CD14^+^ cells). Data are representative of three (blood monocytes and lung macrophages) and 5 (iMϕs) independent experiments. **(F)** iMϕ upregulate HLA-DR in response to LPS. iMϕs were stimulated with 100 ng/ml LPS (LPS) or left unstimulated (control), HLA-DR expression was analyzed 24 h later. Representative results of one out of three independent experiments (Mann-Whitney test; the differences in the mean fluorescence intensity, *p* < 0.05).

On CD14/CD16 dotplots, TB-lung macrophages (CD14^+^ gated lung cells) appeared as one scattered CD14^+^CD16^+^ population composed of a continuum of cells expressing CD14 and CD16 at variable levels (Figure 3A). Compared to blood monocytes that contained CD16^+^ and CD16^−^ subpopulations, TB-lung CD14^+^ cells were homogeneously CD16^int^ (Figure 3B). Other differences between TB-lung macrophages and blood monocytes included a lower expression of CD11b and a higher expression of CD206 by the former (Figures 3C,D). Of note, similar patterns of CD16, CD11b and CD206 expression and similar differences from blood monocytes were displayed by iMϕs (Figures 3B–D).

The expression of HLA-DR, in contrast, differed between TB-lung macrophages and iMϕs, as the former were HLA-DR^hi^, and iMϕs were HLA-DR^dim^ (Figure 3E). This could be due to the fact that TB-lung macrophages were isolated from the infected lungs and thus were activated. Indeed, iMϕs were generated in the absence of inflammatory stimuli in their microenvironment. It is worth noting that the lack of inflammatory microenvironment distinguished iMϕs not only from TB patient-derived macrophages, but also from cells derived from healthy donors, as even in healthy individuals circulating monocytes are exposed to various stimuli (e.g., cytokines and other soluble factors). In line with this, the level of HLA-DR expression by iMϕs was lower not only compared to TB-lung macrophages, but also compared to monocytes/MDMs isolated from the blood of healthy donors. To examine whether iMϕs were able to upregulate HLA-DR in response to inflammation, we stimulated them with LPS. As expected, LPS-stimulated iMϕs significantly increased HLA-DR expression, supporting cell capacity to upregulate HLA-DR in inflammatory conditions (Figure 3F).

To summarize, in phenotypic analysis, iMϕs appeared as differentiated but low-activated/low-polarized cells that differed from blood monocytes, displayed general similarities with MDMs and shared several phenotypic traits with macrophages isolated from the infected human lung.

### iMϕs Co-produce Pro- and Anti-inflammatory Cytokines

**Figure 4:**
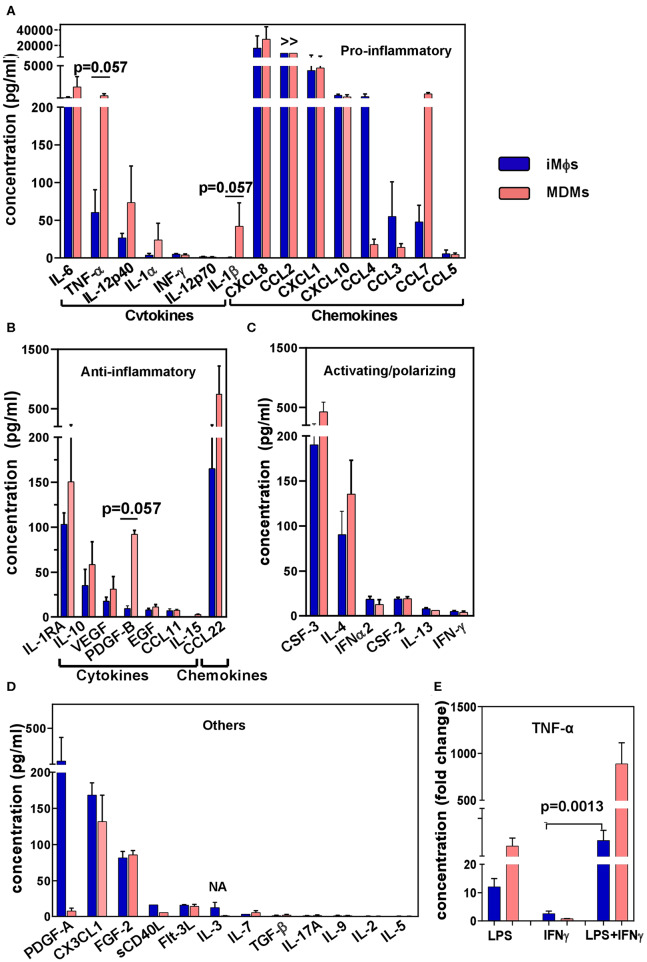
Cytokine secretion profile of iMϕs and MDMs. Supernatants were obtained from iMϕ (blue) and MDM (pink) cultures and analyzed in 41-plex multiplex assay. Results from three independent differentiation experiments were normalized for cell numbers, and are expressed in pg/ml (mean±SEM). **(A)** Pro-inflammatory cytokines. **(B)** Anti-inflammatory cytokines. **(C)** Cytokines involved in macrophage activation/polarization. **(D)** Other factors. >>, factor produced at levels exceeding the upper threshold value of the assay (10 000 pg/ml). **(E)** TNF-α production by iMϕs and MDMs stimulated with LPS, IFN-γ or both. Results show fold increase in TNF-α production by stimulated compared to unstimulated cells (mean ± SEM of three independent experiments). **(A,B)** Mann-Whitney test. **(E)** Kruskal-Wallis test with Benjamini-Hochberg correction; FDR set at *q* = 0.05.

To analyze the iMϕ secretion profile, supernatants were collected from iMϕ cultures and analyzed in a 41-plex cytokine assay (Figure 4). iMϕ supernatants contained both pro- and anti-inflammatory cytokines. Among pro-inflammatory cytokines (known as M1-associated), IL-6, CXCL8, CCL2, CXCL1, CXCL10, and CCL4 were most abundant. However, IL-1β and IL-12 (IL-12p40 and IL12-p70), two main M1-associated pro-inflammatory factors, were found at very low levels (Figure 4A).

Anti-inflammatory factors (known as being M2-associated) secreted by iMϕs included IL-1RA, IL-10, VEGF and CCL22 (Figure 4B). Although the levels of their production were lower compared to many pro-inflammatory cytokines, the IL-10/IL-12p70 ratio was >20. Among cytokines that have been associated with macrophage polarization, CSF3 (G-CSF) and IL-4 were secreted at higher levels compared to CSF2 (GM-CSF), IL-12 and IFN-γ (Figures 4A,C).

A comparison of the iMϕ secretory profile with that of MDMs showed their general similarities. A tendency toward a lower level secretion of TNF-α and IL-1β by the former was detected (p = 0.057), suggesting a less inflammatory status of iMϕs. However, the differences were most probably due to individual variations between the cells derived from genetically different donors (57).

Macrophages are plastic cells that readily respond to environmental stimuli. To assess the ability of iMϕs to react to environmental changes we stimulated iMϕs with LPS, IFN-γ or both, and evaluated the levels of TNF-α in culture supernatants in ELISA. LPS induced a 13.5 fold increase in TNF-α secretion. IFN-γ did not induce a TNF-α response when used as a single stimulus, but significantly promoted TNF-α production when added together with LPS (Figure 4E). This pattern of reactivity was also displayed by MDMs (Figure 4E); it is characteristic for macrophages in general and has been associated with the ability of IFN-γ to suppress feedback inhibitory components of TLR responses (77). Thus, iMϕs displayed a typical pattern of macrophage reactivity to LPS and IFN-γ.

### iMϕs Are Phagocytic and Able to Restrict *Mtb* Growth *in vitro*

A characteristic feature of macrophages is their ability for phagocytosis and antibacterial response. Several previous studies demonstrated that macrophages derived from pluripotent stem cells are able to phagocytose fluorochrome-conjugated beads and bacteria (35, 44, 46). In contrast, the ability of pluripotent stem cell-derived macrophages to restrict bacteria growth remained under-studied.

**Figure 5:**
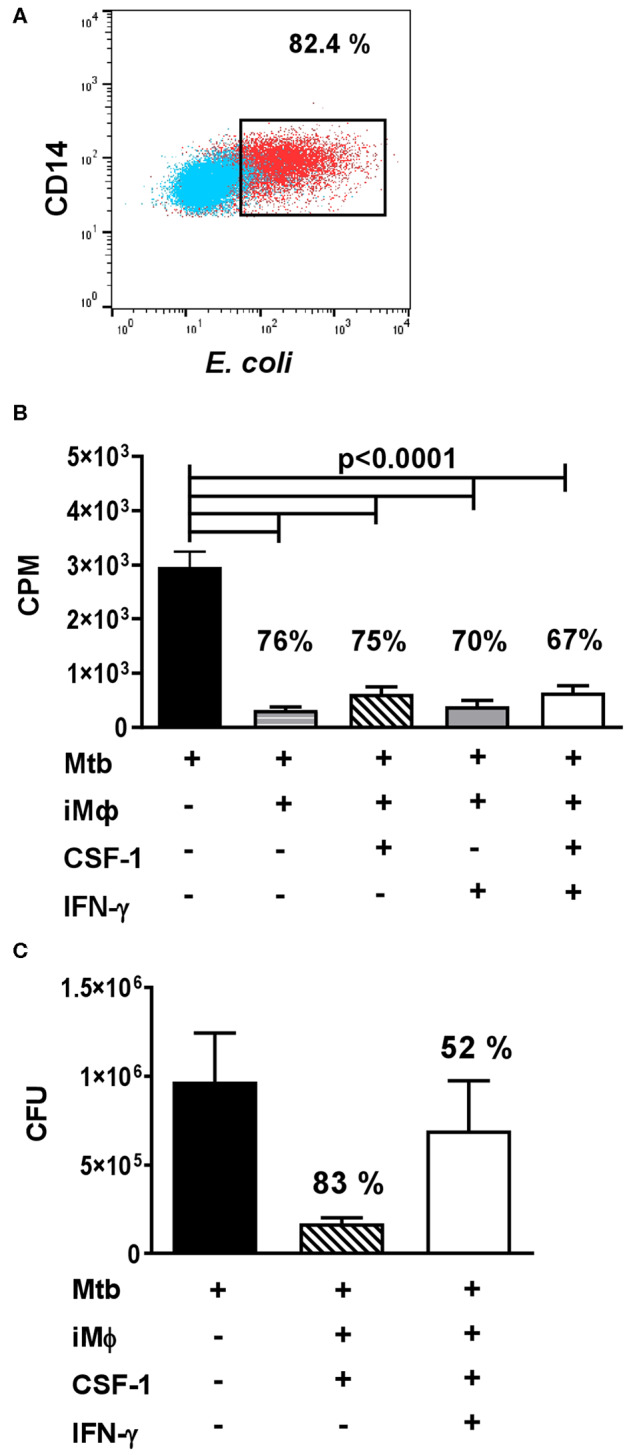
iMϕs exhibit phagocytic and antibacterial activity. **(A)** iMϕs are phagocytic. iMϕs were cultured with FITC-labeled *E.coli* from Phagotest™ kit (BD Bioscience). Blue, control (0°C); red, experiment (37°C). **(B,C)** iMϕs inhibit *Mtb* growth *in vitro*. iMϕs were cultured with *Mtb* at the multiplicity of infection of 5 in the presence or absence of CSF1 or/and IFN-γ. Three days later, *Mtb* growth was assessed in [3H]-uracil uptake assay **(B)** or by plating cell lysates on Dubos agar and enumerating *Mtb* CFUs 21 days later (**C**, confirmation of data presented in **B**). Figures on the graphs indicate the percent of *Mtb* growth inhibition. The results of 5 **(B)** or 2 **(C)** independent experiments are shown. Mean±SEM, Kruskal-Wallis test with Benjamini-Hochberg correction; FDR set at *q* = 0.05.

In our analysis, iMϕs readily engulfed FITC-labeled *E.coli* in a Phagotest™ assay (Figure 5A). To examine antibacterial capacity of iMϕs, we cultured them with live *Mtb* at the multiplicity of infection of 5. *Mtb* growth was assessed using a [3H]-uracile uptake assay and by direct quantification of *Mtb* CFUs in the cultures (78). In both assays, iMϕs inhibited *Mtb* growth by >75% (Figures 5B,C). Similar results were obtained using iMϕs derived from iMA and KYOU iPSC cell lines.

IFN-γ is known to activate macrophage antibacterial function. Surprisingly, we found that IFN-γ did not increase the mycobactericidal activity of iMϕs (Figures 5B,C). We even detected a tendency toward an increased *Mtb* growth in the presence of IFN-γ (Figure 5C). It is important that previous analyses showed that IFN-γ can enhance *Mtb*-triggered necrosis of human macrophages (79). In our study, a decrease in the viability of *Mtb*-infected iMϕs cultured in the presence of IFN-γ was also observed, when the cultures were examined under the microscope (data not shown). Interestingly, in our previous study performed in a mouse model of tuberculosis, IFN-γ increased the mycobactericidal capacity of peritoneal macrophages, but failed to increase the mycobactericidal capacity of lung macrophages (78). Thus, our results were in line with other reports and a poor responsiveness of lung macrophages to this cytokine obtained in other models.

## Discussion

In this article, we aimed to characterize the phenotypic, secretory and antibacterial properties of iMϕs within the framework of one study and to compare iMϕs with monocytes/macrophages persisting *in vivo*.

In phenotypic analysis, iMϕs appeared as CD14^+^CD45^+^CD11b^+^CD64^+^CD16^int^CD195^+^ cells. The expression of common leukocyte (CD45), myeloid (CD11b, CD64) and mature macrophage (CD16, CD195) markers by iMϕs corresponds well to the results of other studies (35, 37, 39, 40).

An intriguing question is a question on the similarities of iMϕs to and their differences from monocytes/macrophages persisting *in vivo*. Some authors reported that iMϕs are comparable to blood monocytes (36, 38, 40, 69, 80). However, others argued in favor of a better similarity of iMϕs to yolk sac or fetal liver derived and tissue resident macrophages (34, 35, 43, 46). The phenotypic comparison of iMϕs and blood monocytes/MDMs performed in our study revealed general similarities between the cells, but also identified differences in the level of the expression of several markers. The differential expression of some markers (e.g., CD11b, CD86) was likely due to the inter-individual variations of cells derived from genetically different donors (57). However, a low-level expression of HLA-DR and the pattern of CD14/CD16 co-expression seem to reflect the characteristic features of iMϕs. Previously, other authors reported the expression of MHC class II molecules by iMϕs, but did not point to a low level of their expression. Our analysis of the published data shows that in these studies, the levels of MHC class II expression by iMϕs were low, which is in line with our results (39, 41, 81). Furthermore, a low expression of genes of the human leukocyte antigen system by un-stimulated iMϕs was found at transcriptomic level (36). This suggests that the HLA-DR^dim^ phenotype is a peculiar feature of iMϕs and characterizes them as low-activated cells.

Based on the co-expression of CD14/CD16, blood monocytes fall into three well-known subsets, CD14^+^CD16^−^, CD14^+^CD16^+^, and CD14^low^CD16^+^ (82). In our study, all three subsets were identified in blood monocytes; MDMs were generally CD14^+^CD16^+^ with a small percentage of CD14^+^CD16^−^ cells; iMϕs and lung macrophages were almost exclusively CD14^+^CD16^dim^. One can assume that the co-expression of CD14 and CD16 is a characteristic feature of all macrophages, not only iPSC-derived ones, and that this feature distinguishes them from monocytes, which are less mature. However, the levels of CD16 expression by iMϕs were lower compared to MDMs; a small proportion of CD14^+^CD16^−^ cells present within MDMs was absent within iMϕs; finally, iPSC-derived cells expressed the CD14^+^CD16^dim^ phenotype starting from the early stages of their differentiation (i.e., at the level of monocyte-like cells, iMCs). Thus, it is likely that the CD14^+^CD16^dim^ phenotype is not due to a “more differentiated” state of iMϕs compared to blood monocytes and rather represents a characteristic feature of iMϕs. In confirmation of that, one previous study reported that embryonic and fetal monocytic cells differentiate to CD14^+^CD16^+^ macrophages without going through the CD14^+^CD16^−^ stage (35), a feature that, according to our data, unites embryonic macrophages and iMϕs.

Macrophages are plastic cells that respond to external stimuli by changing the pattern of gene and protein expression and acquiring different activation states. To characterize macrophage reactivity, two types of stimuli, LPS/IFN-γ (“M1-polarizing”) and IL-4 (“M2-polarizing”) are widely used. The stimuli induce distinct activation programs characterized by different gene expression profiles, secretomes and phenotypic traits (26, 53–55). At the phenotypic level, several markers were associated with IFN-γ/LPS and IL-4 stimulated macrophages. Although it is currently understood that macrophage response to LPS/IFN-γ and IL-4 does not reflect the whole diversity of macrophage activation states and that the markers of stimulated macrophages detected in different studies do not fully correlate with each other (26, 55, 58, 65), macrophage phenotyping is still used to assess cell bias toward the pro- (“M1”) or anti- (“M2”) inflammatory type. In our study, iMϕs appeared as CD80^dim^CD86^dim^HLA-DR^dim^CD195^+^CD163^+^CD206^+^ cells, i.e., they were non-polarized or were slightly biased toward the expression of an anti-inflammatory phenotype. The latter follows from a higher expression level of CD163 and CD206 compared to CD80 and CD86, the upregulation of CD163 and CD206 (but not CD80 and CD86) during the process of iMC differentiation into iMϕs and the pattern of cytokine secretion (see below). The conclusion corresponds well to the use of CSF1 for iMϕ generation, as CSF1 was reported to bias macrophages to acquire anti-inflammatory characteristics (83), and allows considering iMϕs as CSF1-polarized macrophages.

In line with their unpolarized/low-polarized surface phenotype, iMϕs displayed a mixed secretory activity, which is exemplified by the production of pro- (i.e. IL-6, CXCL8, CCL2, CXCL1, CXCL10, CCL4) and anti- (i.e., IL-1RA, IL-10, CCL22) inflammatory cytokines. Notably, IL-12p70 and IL-1β were produced by iMϕs at very low levels, and the IL-10/IL12p70 ratio was >20. The data, for the first time, characterize iMϕs as low-polarized “naïve-like” macrophages with a slight bias toward the anti-inflammatory activity. This further unites iMϕs with embryonic macrophages and macrophages persisting *in vivo*. Indeed, one previous study reported the M2 bias of macrophages derived from human ESCs and fetal liver (35). *In vivo*, macrophage populations are not fully polarized and rather represent a spectrum of states between the M1 and M2 polarities (64, 84). Macrophages persisting in different pathological conditions were also reported to display mixed M1/M2 surface or secretory phenotypes (52, 85, 86).

Several recent studies have demonstrated that lung-associated macrophages are phenotypically and functionally heterogeneous (87–89). iMϕs generated in our study were reminiscent of CD206^+^ population of lung interstitial macrophages described by Schyns and co-authors (89). The similarities between the two types of macrophages include a high-degree of vacuolation, large size, the expression of CD206, low-level expression of MHC class II, and the secretion of pro-inflammatory along with anti-inflammatory cytokines.

Macrophages readily change their phenotype and secretory profile following their stimulation. Our data have documented the ability of iMϕs to up-regulate HLA-DR and to produce TNF-α in response to inflammatory stimuli, such as LPS. Together with our other observations, this suggests that iMϕs represent a valuable model to analyse the response of “naïve-like” macrophages to different stimuli. With this respect, it will be important to examine the reactivity of iMϕs to other stimuli. These studies are currently ongoing.

A separate part of our study investigated the antimycobacterial capacity of iMϕs. The phagocytic activity of macrophages derived from pluripotent stem cells was previously shown by several groups (37, 39, 40, 43, 45). In contrast, cell capacity to inhibit pathogens has not been studied until recently. Our data demonstrate that iMϕs: (i) are able to inhibit *Mtb* growth *in vitro*, i.e., that they have the machinery to exhibit antibacterial response; (ii) do not clear *Mtb* completely. The data are in line with recent report on the capacity of iMϕs to kill *P. aeruginosa* (69) and indicate that iMϕs represent a suitable model for investigating fundamental and applied questions of anti-tuberculosis protection including searching for new antituberculosis drugs, as was done recently by Han and co-authors (68).

The current study has some limitations. First, although we directly compared the phenotype of iMϕs with cells persisting *in vivo*, i.e., blood monocytes and lung macrophages, the latter were isolated from TB patients, i.e., they did not recapitulate populations residing in the lung in steady-state conditions. Additionally, iMϕs and blood and lung cells were not isogenic, indicating that some differences between the cells could be due to genetically determined inter-individual variations (57). On the other hand, some differences (i.e., the level of HLA-DR expression and the pattern of CD14/CD16 co-expression) appeared to be steady. Notably, TB-lung macrophages and blood monocytes were derived from the same donors. Thus, the differences between iMϕs and blood monocytes and the similarities between iMϕs and lung macrophages could not be attributed exclusively to the inter-individual variations between the donors. Second, due to biosafety reasons we could not use fluorescence sorting to purify lung macrophages, which precluded the analysis of their secretory and antibacterial activity. Finally, it is understood that transcriptomic analysis of iMC and iMϕ populations would help to obtain more detailed information on the biology of these cells. Such experiments are currently ongoing. In this study we focused specifically on the phenotypic and functional characterization of iMϕs, which is significant for understanding iMϕ immunology.

To summarize, our results characterize iMϕs as differentiated, low-activated low-polarized cells that exhibit multifarious functional activities, including antibacterial properties. iMϕs differ from blood monocytes, are comparable to MDMs and lung-associated macrophages and represent a valuable model for studying macrophage functioning, especially, for modeling and modulating macrophage-pathogen interactions.

## Data Availability Statement

The datasets generated for this study are available on request to the corresponding author.

## Ethics Statement

The studies involving human participants were reviewed and approved by IRB#1 of the Central Tuberculosis Research Institute. The patients/participants provided their written informed consent to participate in this study.

## Author Contributions

IL: Initiation of the study, study design, supervision, and manuscript writing. TN, TG, and YS: Performance of the majority of the experiments. EG, YS, and ED: iPSC differentiation. GK: Lung cell isolation and cytospin analysis. TN and AN: Experiments involving *Mtb*. IL, TN, and TG: Data analysis and interpretation. TN and TG: Manuscript editing and figure preparation.

## Conflict of Interest

The authors declare that the research was conducted in the absence of any commercial or financial relationships that could be construed as a potential conflict of interest.

## Abbreviations

CFUs: colony forming units
EBs: embryoid bodies
iPSCs: induced pluripotent stem cells
iMCs: monocyte-like cells derived from iPSCs
iMϕs: macrophages derived from iPSCs
MDMs: blood monocyte-derived macrophages
*Mtb*: *Mycobacterium tuberculosis*
TRMs: tissue resident macrophages
TB: tuberculosis.
